# Health Lectures

**Published:** 1858-02

**Authors:** 


### HEALTH LECTURES.

It is said that Messrs. Douglass & Sherwood of this city, who employ three hundred girls in their shirt manufactory, have instituted a course of lectures, during working hours, on Mental, Moral, and Physical Culture, and the best means of improving their health.

As a means of solid, useful instruction in physics, morals, or religion, "popular lectures" are a nuisance, for two principal reasons. 1. Lecturers will strain the truth for the sake of a point. 2. They too often sacrifice religion and its ministers to make a hit, said "hit" seldom failing of rapturous applause.

A more efficient and more permanent method of benefiting operatives would be, the distribution of a few dollars worth of our Journal among them every month; where the articles can be read, thought of, and re-read, and then laid away for after reference. A "lecture" on physiology or hygiene is too stiff and formal for a girl at work; and it is very certain that either the lecture or the work will suffer.

The evident benevolence of Messrs. D. & S. merits suggestions which may further the humane objects they have in view.

